# The effect of psychological distance on intertemporal choice of the reward processing: an eye-tracking investigation

**DOI:** 10.3389/fpsyg.2024.1275484

**Published:** 2024-01-31

**Authors:** Yujie Li, Xiaoyi Chu

**Affiliations:** Department of Health Management, Shandong Drug and Food Vocational College, Weihai, China

**Keywords:** psychological distance, intertemporal choice, construal level, timporal distance, social distance, probability distance

## Abstract

This study employed eye-tracking technology to investigate how varying dimensions of psychological distance–temporal, probability, and social–affect intertemporal choice. Across three experiments, participants were asked to select between two intertemporal options while their eye movements were monitored. Findings revealed inconsistent impacts of different psychological distances on intertemporal decision-making. Increased temporal and social distances led to a preference for larger delayed rewards (Studies 1 and 3), whereas an increase in probability distance did not significantly alter choice preferences (Study 2). The research also highlighted a general pattern in information processing; as psychological distance widened, participants showed a tendency toward dimension-specific processing in making intertemporal decisions.

## 1 Introduction

### 1.1 Intertemporal choice

Intertemporal choice is the process whereby people make judgments and choices by weighing gains or losses that occur at different points in time (Frederick et al., 2002). This is no strange matter in the lives of individual, for it bears a heavy influence on personal matters ranging from everyday consumption to educational pursuits and healthcare practices. It also bears upon the loftier aspects of national governance and public policy formulation (Loewenstein and Thaler, 1989; Strulik, 2021). Intertemporal decision-making in the lab has typically involved time and amount: “Sooner and Smaller, SS” and “Later and Larger, LL.” The latter option provides more or better results at the cost of time (Weber et al., 2007; Read et al., 2013, 2017). Within the diverse and complex landscape of intertemporal decision-making, a multitude of factors come into play. Decisions may be made not only for personal circumstances but also for the guidance of others. An event’s temporal unfolding, geographical context, and the magnitude of its associated probabilities can all shape the intertemporal choices’ trajectory.

### 1.2 Psychological distance and level of construal

Psychological distance refers to the extent to which a stimulus (object or event) is removed from the perceivers’ direct experience (Trope et al., 2007). As an event can be removed from direct experience along multiple dimensions, psychological distance has multiple dimensions as well. Generally, distance occurs in four dimensions: temporal distance, spatial distance, social distance, and probability distance. As regards time, distant things occur in the past or in the future; as regards space, distant things occur in remote locations; as regards interpersonal relationships, distant things happen to others who have nothing to do with oneself; as regards possibility, distant things are almost impossible to occur (Liberman et al., 2002; Trope and Liberman, 2010; Maglio et al., 2013b). The perception of psychological distance is subject to individual variability, influenced by factors such as personal experience, cultural background, emotional state, personality traits, and the relevance of the event or object to the individual. These diverse influences underscore the nuanced and complex nature of psychological distance perception.

According to the construal level theory (CLT), we can explain the mechanisms that trigger assessment, prediction, and decision-making behavior by linking psychological distance to construal level (Nussbaum et al., 2003; Fiedler, 2007; Trope and Liberman, 2010; Adler and Sarstedt, 2021). People’s response to social events depends to a large extent on how he or she mentally construes the event (Liberman et al., 2002). The low-level constructs involve a relatively unstructured, contextualized representation of an event that includes incidental and subordinate features. The high-level constructs are abstract, schematic, decontextualized representations that extract the gist of the available information (Trope and Liberman, 2010). Studies indicated that the various dimensions of psychological distance operate interchangeably, casting a similar influence on how objects are constructed (Bar-Anan et al., 2006; Pronin et al., 2008; Liberman and Förster, 2009; Trope and Liberman, 2010; Fiedler et al., 2012; Chen and He, 2014). People mentally construct objects that are psychologically near in terms of low-level construal, whereas they construe events at a distance in terms of high-level construal (Liberman and Trope, 1998; Liberman et al., 2002; Bar-Anan et al., 2006; Wakslak et al., 2006; Fujita et al., 2006b; Trope et al., 2007; Liviatan et al., 2008). Individuals with different construal level have different focus on attributes and varying perspectives in decision making (Fujita and Roberts, 2010). An increase in psychological distance tends to lead features associated with high-level construal receive greater weight during decision-making evaluations (Liberman and Trope, 1998; Trope and Liberman, 2010).

Some research findings support this viewpoint, focusing on a variety of construal aspects and examining a variety of behaviors (Trope and Liberman, 2000; Sagristano et al., 2002; Fujita et al., 2006a; Kim et al., 2008; Maglio et al., 2013a). There are accumulating evidence that the same principle of the CLT applies to all dimensions of psychological distance (Fiedler et al., 2012). For instance, when considering goal-directed action, cause is a high-level feature of an event compared to consequence, since the consequences depend on the causes but not the reverse. Based on multiple manipulations of psychological distance, Rim et al. (2013) suggested that temporal and social distances may be influenced by the same general principle. Results revealed that the increase in psychological distance led to a greater tendency to focus on the underlying causes (vs. consequences) yielded by their actions. Similarly, desirability concerns are related to its goal value, while the action’s feasibility concerns are related to its specific method of achieving that goal. Accordingly, desirability concerns are features of events that are more high-level relative to consequence in our conceptualization. Studies indicated that people emphasize desirability concerns more than feasibility concerns as the probability distance increases (Todorov et al., 2007). Moreover, this effect is not limited to a single dimension of psychological distance, but also to temporal, spatial, and social distances as well (Trope and Liberman, 2010; Mann et al., 2013; Maglio et al., 2013b).

### 1.3 Psychological distance and intertemporal choice

Much research has been conducted to determine the effect of psychological distance on intertemporal choices, but is still a matter for intense and controversial debate. In intertemporal decisions based on monetary experimental tasks, the delay time of the options is considered as a secondary, low-level construal characteristic, whil the value magnitude of the options is regarded as a primary, high-level construal feature (Fujita et al., 2006a). Thus, on the basis of the CLT, an increase in psychological distance will lead decision-makers to favor delayed options with greater monetary value in intertemporal choices.

Some results still favor the view of the CLT. For instance, the immediacy effect indicated that people often exhibit a preference for the immediate, smaller reward, over a larger reward that is delayed. As the time distance between both options increases, the more individuals tend to choose the delayed large options (Frederick et al., 2002; Weber et al., 2007; Read et al., 2013). In addition, there were studies indicated that the inclusion of probability distance will reduce the premium people place on immediate rewards (Keren and Roelofsma, 1995; Weber and Chapman, 2005; Chen and He, 2014). People make decisions differently for others versus themselves. When making decisions for strangers, individuals were more likely to choose the large delayed reward than the immediate small reward (Albrecht et al., 2011; Kim et al., 2013; Piva et al., 2019). With respect to spatial distance, participants in the distal condition were more likely to choose the larger, temporally delayed reward than those in the proximal condition (Maglio et al., 2013b).

However, on the other hand, there were studies suggested that different psychological distance may not be interchangeable in the psychological impact on preference. In intertemporal matching tasks, Öncüler (2000) found that the external risk will increase rather than decrease the degree of discounting future, which indicated that the probability distance may reduce participants’ patience during intertemporal choices. Using a simulated intertemporal choice scenario, Anderson and Stafford (2009) found that subjects are less patient when they are faced with high risky (probability distance) payoffs. In an extension of these findings, Sun and Li (2010) examined the role of temporal distance and probability distance in intertemporal decision-making. In two experiments, it was found that probability distance may increase the extent of small options in the near future, while time delays have the opposite effect. In addition, studies indicated that doctors made more conservative decisions for their patients than for themselves (Garcia-Retamero and Galesic, 2012). In terms of the CLT, these phenomena are difficult to explain.

### 1.4 Eye-tracking method and intertemporal choice

Some researchers raised the possibility that heterogeneity in methods may affect experiment results (Weber and Chapman, 2005; Hardisty and Pfeffer, 2017). The research methodology may hinder our ability to understand this issue. The discrepancy between existing experimental results on intertemporal decision making with risk may be the sensitivity of the elicitation procedure to the degree of discounting the future (Sun and Li, 2010). Some evidence suggests that the outcome-based paradigm comes with inherent limitations, making it difficult to offer more direct and objective evidence concerning the correlation between information inputs and outputs within the decision-making process (Schulte-Mecklenbeck et al., 2011).

As one of the process-based paradigms, using eye-tracking research methods can effectively mitigate such impact. The eye-tracking variable (i.e., number of fixations, fixation duration, search metric) are critical indices in decision-making outcomes (Fiedler et al., 2012; Reeck et al., 2017; Amasino et al., 2019). By understanding and analyzing these indices, we can provide more additional procedural evidence. Eye-tracking studies of intertemporal decision making have focused on the causal relationship between process characteristics of decision making and choice preferences (Fisher, 2021). For instance, studies have found that manipulating dimension-based eye movements can impact intertemporal decision-making behavior, underscoring the important role that dimension-based information processing plays in intertemporal decision-making (Franco-Watkins et al., 2016; Liu et al., 2023).

In summary, the relationship between psychological distance and intemporal choices is still unclear. Although the CLT contributes to our understanding of intertemporal decision-making, it still needs further testing. Used eye-tracking technology and three parallel experiments, we sought to: (1) Examine the effect of psychological distance (temporal distance, social distance and probability distance) on the intertemporal choice. (2) Examine participants’ decision process characteristics under the different psychological distance conditions.

## 2 Experiment 1

### 2.1 Participants

Sample sizes were calculated using G*Power software (v.3.1.9.2) (Faul et al., 2007). For the mixed repeated measures ANOVA applied in this study, the total sample size required to predict the level of statistical power needed to reach 95% was at least 76 at a significance level of *α* = 0.01 and a medium effect (*F* = 0.25). Therefore, a total of 80 participants (44 female; Mage = 19.48, SDage = 1.12) were recruited from a college. All of them have normal or corrected-to-normal vision. The experiment was approved by the researchers’ University Ethical Advisory Committee. All the participants provided written consent before the experiment. To further incentive their cooperation, participants received a flat payment of 30 CNY plus between 20 and 60 CNY. Payments to those who chose delayed options were not delayed. After the experiment, we debriefed all participants to uphold ethical standards and ensure their psychological well-being. The structured debriefing included explaining the study’s purpose, disclosing any deceptions, and addressing participants’ concerns.

### 2.2 Apparatus

The experiment was conducted in a quiet dedicated eye-tracking laboratory. We used the TobiiX120 eye tracker at a sampling rate of 120 to collect all eye tracking data. The experimental stimuli were presented on a 19inch LCD display (60 Hz refresh rate, 1,280 × 1,024 screen resolution). Participant sat approximately 50 cm in front of the monitor and entered their choices via a keyboard. Their horizontal viewing angle was 28°, and the vertical viewing angle was 21°. A standard nine-point calibration scheme was used and if gaze error was more than 1 degree during validation a recalibration was conducted. The background color of the calibration screen and all instructions presented to the participants were set to the luminance of the experimental trials to ensure that stable data was recorded (Madsen et al., 2021).

### 2.3 Materials and procedure

After giving their consent, participants were informed about the experiment and given a brief explanation of the apparatus. The experimental guidance informed the participants that we are preparing for a lottery activity and want to investigate which prize setting would be more attractive. Participants were required to complete a control task that consisted of 16 trials. In each trial, they had to choose between two bonus options: “Sooner and Smaller, SS” and “Later and Larger, LL.” The materials (psychological distance and intertemporal choice) used in this study were similar to those used in previous study (Chen and He, 2014; Zhou et al., 2019). For instance, they could opt for an immediate 450 CNY or a larger sum of 600 CNY available a month later. The near-temporal distance group was told that the activity is announced to take place on the same day, whil in the far-temporal distance group, the activity is announced to take place 30 days later. To validate the manipulation of psychological distance in the experiment, we pre-tested the temporal distance manipulation with 114 participants (*M*age = 19.84, SDage = 0.92). The participants reported experiencing a greater psychological distance during the far-temporal distance condition (*M* = 5.43, SD = 1.21) compared to the near-temporal distance condition (*M* = 2.52, SD = 1.12), *t* (113) = 18.54, *p* < 0. 001, Cohen’s d = 1.74, 95% CI [1.44, 2.03].

**Figure 1:**
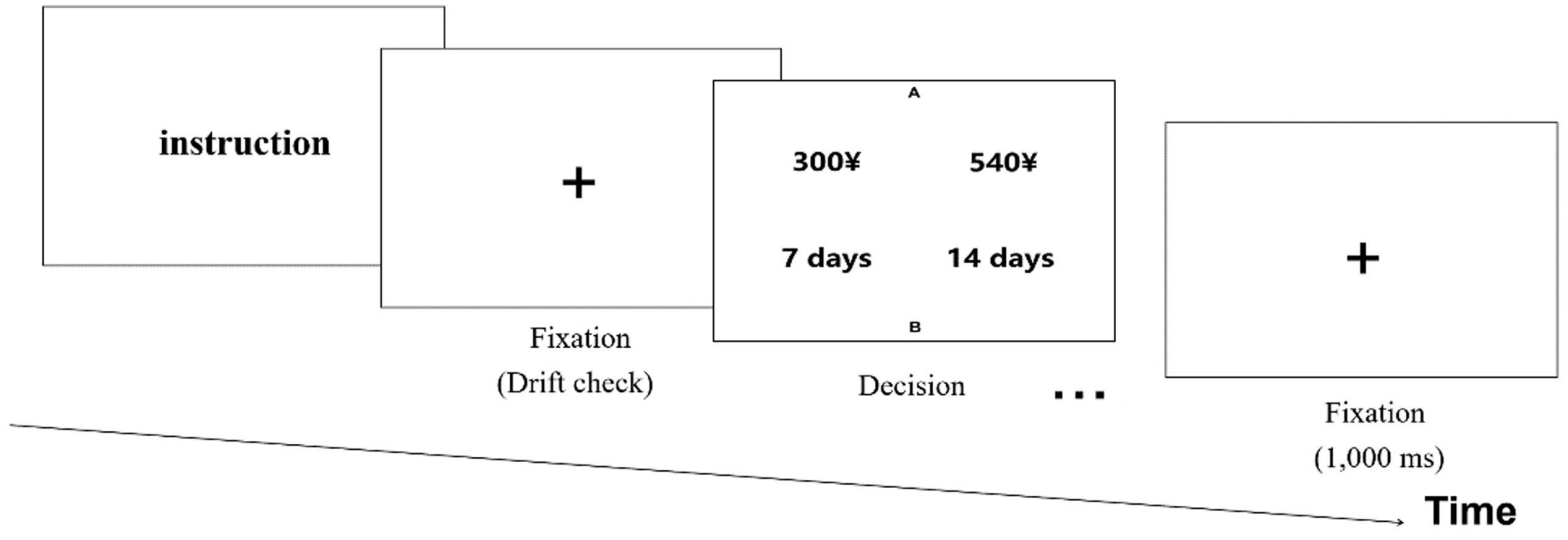
Trail procedure and time in experimental tasks.

At the beginning of each trail, a fixation disk was presented, and a drift correction was performed to ensure the accuracy of the eye movement record. When fixation on that disk was registered, the participants were asked to press F and J on the keyboard to make their choices. After each participant responded, a 1,000 ms interval was shown before the next trial began. To avoid the effect of option presentation on the information-search process, we counterbalanced the position of each pair in each top/bottom or left/right position. The (horizontal/vertical) center-to-center distance between any two values was greater than 5°. As a preliminary preparation for the formal experiment, participants have to read the instructions and complete four practice trials. They did not report any lags or difficulties with revealing attributes (Figure 1).

### 2.4 Measures of eye movement data

The eye movement data were recorded and pre-processed by Tobii Studio software (version 3.0). Four 350 × 250 pixels rectangular regions of interest (AOIs) were defined, covering time and amount in each of the two options. The visual angle of each AOI subtended 12.26° by 7.04°. Fixations were described as periods of a relatively stable gaze between two saccades. Specifically, we analyzed the following process measures:

#### 2.4.1 Duration of fixations

The fixation duration is a reliable parameter for levels of attention and consumption of cognitive resources (Glöckner and Herbold, 2011). If the decision-making involves compensatory rules, it may entail a cautious calculation process (Horstmann et al., 2009; Orquin and Loose, 2013), and the average fixation duration should be relatively longer (Zhou et al., 2019). On the other hand, if it is not based on compensatory rules, then it might not involve such cautious calculation.

#### 2.4.2 Search metric index

To evaluate the overall pattern of information acquisition, we applying an search metric calculation (Böckenholt and Hynan, 1994). The index describes an individual’s preference for alternative-wise or dimension-wise processing. It has been widely used in eye-movement studies for decision making (Su et al., 2013; Schulte-Mecklenbeck et al., 2017; Zhou et al., 2019). It is calculated as follows:


$$SM=\frac{\sqrt{N}\left[\left(\mathrm{AD}/N\right)\left(r_{a}-r_{d}\right)-\left(D-A\right)\right]}{\sqrt{A^{2}\left(D-1\right)+D^{2}\left(A-1\right)}}$$

where A and D denote the number of alternatives and the number of dimensions, respectively; *r*_a_ and *r*_d_ denote the number of alternative-based saccades and dimension-based saccades, respectively, and N denotes the number of total saccades. An *SM*>0 indicate that the individual’s dominant processing pattern is alternative-based, and vice versa, it is more dimension-based (Schulte-Mecklenbeck et al., 2017).

#### 2.4.3 Attentional processing and choice

To examine whether there was differential processing in selection bias, we applying the calculation (Krajbich and Rangel, 2011; Franco-Watkins et al., 2016; Amasino et al., 2019; LIU et al., 2023) based on fixation durations as follows:


$$SB_{a}=\frac{\left(AOI_{\mathrm{sooner}}+AOI_{\mathrm{smaller}\;\mathrm{money}}\right)-\left(AOI_{\mathrm{later}}+AOI_{\mathrm{larger}\;\mathrm{money}}\right)}{\mathrm{Total}\;\mathrm{AOIs}}$$

An *SBa*>0 indicate that the extreme bias to only immediate option parameters, and vice versa, it indicates extreme bias to only the delayed option parameters. An *SBa* score of 0 indicates no specific bias to either option (Franco-Watkins et al., 2016).


$$SB_{d}=\frac{\left(AOI_{\mathrm{sooner}}+AOI_{\mathrm{later}}\right)-\left(AOI_{\mathrm{smaller}\;\mathrm{money}}+AOI_{\mathrm{larger}\;\mathrm{money}}\right)}{\mathrm{Total}\;\mathrm{AOIs}}$$

An *SBd*>0 indicate that the extreme bias to the time attribute, and vice versa, it indicates extreme bias to only the amount attribute.

### 2.5 Results

A t-test was conducted with the participants’ grouping as the independent variable and the proportion of choosing the SS option (get a small amount of money in the nearer future) as the dependent variable. The proportion of the subjects choosing the SS option in the near-temporal distance condition (*M* = 0.78, SD = 0.14) was significantly higher than in the far-temporal distance condition (*M* = 0.53, SD = 0.19), *t* (78) = 6.61, *p* < 0. 001, Cohen’s d = 1.48, 95% CI [0.97, 1.98] (Table 1).

**Table 1:** Descriptive statistics of key variables of Experiment 1 (*M* ± SD).

Variable	Near-temporal distance	Far-temporal distance	*t*
Fixation duration	2.16 ± 0.96	2.50 ± 1.57	−1.72
*SM*	0.21 ± 0.48	−0.14 ± 0.39	6.61**
*SB_a_*	0.09 ± 0.16	0.01 ± 0.18	1.4
*SB_d_*	−0.16 ± 0.25	−0.08 ± 0.24	−1.12

∗∗*p* < 0.01.

In this study, we excluded trials shorter than 200 milliseconds (10trials, approximately 0.78%) from the analysis based on the prior study (Zhou et al., 2019). The t-test was performed with the duration of fixation, *SM*, and *SB* values as dependent variables, respectively. The SM values for near-temporal distance condition are significantly greater than those for far-temporal distance condition. *t* (78) = 6.61, *p* < 0. 001, Cohen’s d = 0.79, 95% CI [0.33, 1.24]. A one-sample *t*-test was performed to compare the SM values of the two conditions with 0. The results showed that the SM value for the near-temporal distance condition was significantly more than 0 (*t* (39) = 2.74, *p* < 0.01, Cohen’ s d = 0.43, 95% CI [0.11, 0.76]), whil the far-temporal distance condition was significantly less than 0 [*t* (39) = −2.21, *p* < 0.05, Cohen’ s d = −0.35, 95% CI (−0.67, −0.03)]. There was no significant difference in the average fixation duration between the two groups, *t* = −1.72, *p* = 0.09, *Cohen’s d* = −0.38, 95% CI [−0.83, 0.06]. The *SB_a_* and *SB_d_* value difference was also not significant, *t* (78) = 1.4, *p* = 0.17, Cohen’s d = 0.31, 95% CI [−0.13, 0.75]; *t* (78) = −1.12, *p* = 0.24, *Cohen’s d* = −0.26, 95% CI [−0.7, 0.18].

## 3 Experiment 2

### 3.1 Participants

Eighty participants (43 female; *M*age = 20.14, SDage = 0.95) from a university were recruited and randomly divided into a near-probability distance group of 40 and a far-probability distance group of 40.

### 3.2 Materials and procedure

The near-probability distance group was told that the probability of receiving this bonus at the corresponding time was 90%, and the far-probability distance group was told that the probability of receiving this bonus at the corresponding time was only 10%. The questions in the cross-period selection scenario were identical to those in Study 1.

We pre-tested the probability distance manipulation with 114 participants (*M*age = 19.84, *SD*age = 0.92), who reported experiencing a greater psychological distance during the far-probability distance condition (*M* = 5.52, SD = 1.12) compared to the near-probability distance condition (*M* = 2.55, SD = 1.14), *t* (113) = 19.37, *p* < 0. 001, Cohen’s d = 1.81, 95% CI [1.51, 2.11].

### 3.3 Results

The t-test analysis indicated that there was no statistically significant difference between the near-probability distance condition (*M* = 0.63, SD = 0.24) and far-probability distance condition (*M* = 0.61, SD = 0.25), *t* (78) = 0.48, *p* = 0.63, Cohen’s d = 0.11, 95% CI [−0.55, 0.33] (Table 2).

**Table 2:** Descriptive statistics of key variables of Experiment 2 (*M* ± SD).

Variable	Near-probability distance	Far-probability distance	*t*
Fixation duration	2.15 ± 0.94	2.38 ± 1.42	−1.23
*SM*	0.16 ± 0.44	0.20 ± 0.41	−0.25
*SB_a_*	0.08 ± 0.17	0.09 ± 0.16	−1.36
*SB_d_*	−0.14 ± 0.24	−0.18 ± 0.27	0.38

We excluded trials shorter than 200 milliseconds (11trials, approximately 0.85%) from the analysis. The t-test was performed with the duration of fixation, *SM*, and *SB* values as dependent variables, respectively. The *SM* values for the two psychological distance condition was not significant. *t* (78) = −0.25, *p* = 0.8, Cohen’s d = −0.06, 95% CI [−0.5, 0.38]. A one-sample *t*-test was performed to compare the *SM* values of the two conditions with 0. The results showed that the *SM* value for the near-probability distance condition was significantly more than 0 [*t* (39) = 2.73, *p* < 0.01, Cohen’ s d = 0.43, 95% CI (0.11, 0.75)], whil the far-temporal distance condition was significantly more than 0 [*t* (39) = 2.66, *p* < 0.05, Cohen’ s d = 0.42, 95% CI (0.1, 0.74)]. There was no significant difference in the average fixation duration between the two groups, *t* = −1.23, *p* = 0.22, Cohen’s d = −0.28, 95% CI [−0.72, 0.17]. The *SB_a_* and *SB_d_* value difference was also not significant, *t* (78) = −1.36, *p* = 0.18, Cohen’s d = −0.3, 95% CI [−0.74, 0.14]; *t* (78) = 0.38, *p* = 0.71, Cohen’s d = 0.08, 95% CI [−0.35, 0.52].

## 4 Experiment 3

### 4.1 Participants

Seventy-five participants (40 female; Mage = 20.82, SDage = 1.33) from a university were recruited and randomly divided into a near-social distance group of 38 and a far-social distance group of 37.

### 4.2 Materials and procedure

The near-social distance group was told to make a choice between two bonuses awarded for themselves, and the far-social distance group was told that they were making a choice between bonuses for an absent stranger for unspecified reasons. The questions in the cross-period selection scenario were identical to those in Study 1.

We pre-tested the social distance manipulation with 114 participants (*M*age = 19.84, SDage = 0.92), who reported experiencing a greater psychological distance during the far social distance condition (*M* = 5.35, SD = 1.11) than in the near social distance condition (*M* = 2.53, SD = 1.2), *t* (113) = 18.08, *p* < 0. 001, Cohen’s d = 1.69, 95% CI [1.40, 1.98].

### 4.3 Results

The proportion of the subjects choosing the SS option in the near-social distance condition (*M* = 0.69, SD = 0.25) was significantly higher than in the far-social distance condition (*M* = 0.55, SD = 0.2), *t* (78) = 2.66, *p* < 0. 01, Cohen’s d = 0.62, 95% CI [0.15, 1.08] (Table 3).

**Table 3:** Descriptive statistics of key variables of Experiment 3 (*M* ± SD).

Variable	Near–social distance	Far-social distance	*t*
Fixation duration	2.03 ± 0.99	2.03 ± 1.02	−0.13
*SM*	0.35 ± 0.17	−0.27 ± 0.24	7.5**
*SB_a_*	0.10 ± 0.17	0.12 ± 0.18	−0.04
*SB_d_*	−0.20 ± 0.28	−0.18 ± 0.20	−0.4

∗∗*p* < 0.01.

We excluded trials shorter than 200 milliseconds (9trials, approximately 0.75%) from the analysis. The SM values for near-social distance condition are significantly greater than for far-social distance condition. *t* (73) = 7.5, *p* < 0. 001, Cohen’s d = 1.74, 95% CI [1.19, 2.26]. A one-sample t-test was performed to compare the SM value of the two conditions with 0. The results showed that the *SM* value for the near-social distance condition was significantly greater than 0 [*t* (37) = 7.23, *p* < 0.001, *Cohen’ s d* = 1.17, 95% CI (0.75, 1.58)], whil the far-social distance condition was significantly less than 0 [*t* (36) = −3.11, *p* < 0.01, Cohen’s d = −0.51, 95% CI (−0.85, −0.17)]. There was no significant difference in the average fixation duration between the two groups, *t* = −0.13, *p* = 0.89, Cohen’s d = −0.03, 95% CI [−0.48, 0.42]. The *SB_a_* and *SB_d_* value difference was also not significant, *t* (78) = −0.04, *p* = 0.97, Cohen’s d = −0.01, 95% CI [−0.46, 0.44]; *t* (78) = −0.4, *p* = 0.69, Cohen’s d = −0.09, 95% CI [−0.54, 0.38].

## 5 Discussions

### 5.1 The effect of psychological distance on intertemporal choice

This study used eye-tracking technology to systematically examine the effects of temporal distance, probability distance, and social distance on intertemporal choice. In general, participants did not consistently prefer the high-value option LL when psychological distances were increased. As the temporal distance and social distance increased, the proportion of the subjects choosing the SS option in the small distance condition was significantly higher than in the far distance condition. This is consistent with previous research findings (Pronin et al., 2008; Kim et al., 2013; Chen and He, 2014). However, as the probability distance increased, the participants’ selection preferences did not undergo significant changes in this study.

According to the CLT, the link between the proximity of different types of psychological distances and the construal level is similar (Bar-Anan et al., 2006). The more psychologically distant an event is, the more it will be represented at higher levels of abstraction (Trope et al., 2007). With the increase of psychological distance, features associated with high-level construal receive greater weight during decision-making evaluations (Trope and Liberman, 2010). Some aspects of this interpretation have been questioned. Each of these four domains, for instance, has its own motivational and cognitive characteristics, which may lead to multiple pathways for different psychological influences on decision making (Maglio et al., 2013a). Studies indicated that people are more impatient with gambling than with certain outcomes (Frederick et al., 2002; Anderson and Stafford, 2009), they may prefer to choose the fewer immediate rewards due to perceived future uncertainty (even if only implicit) (Andreoni and Sprenger, 2012; Hardisty and Pfeffer, 2017; Xu et al., 2022). This could be one explanation that the impact of different psychological distances on the level of construal is consistent in direction, varying only in the degree of influence. Perhaps the construal level did change when the psychological distance increases, but that the two psychological processes of processing information pushed in opposite directions, canceling each other out and resulting in a null effect.

Furthermore, there were studies indicated that the abundance of information results in a lack of attention (Li et al., 2021). There was a difference in results between adding psychological distance inside or outside the options (Sun and Li, 2010). Switching between information will leave attention residue, which will make it difficult to return to the original task fully focused (Öncüler, 2000; Leroy and Glomb, 2018). Individuals will become less sensitive to differences between information when situations become complex (Li et al., 2021). Research has shown, for instance, that people of different psychological distances make different choices. They exhibit lower sensitivity to time discounting when evaluating delayed options for their friends in comparison to strangers. A possible explanation for this is that people who need to process social distance when making decisions for others consume different cognitive resources (Tang et al., 2021). In the context of this study, the probability distance was presented externally in advance through the guidance. It may lead to an insufficient sensitivity to the assessment of time concerning the results, thereby making the difference of the choices appear smaller.

### 5.2 The eye-tracking index during the intertemporal choice

According to the construal level theory, there will be more comparisons based on the monetary dimension during eye-tracking processes. From the results of the eye-movement index, the subjects in the far-probability distance condition did not show an increase in the depth of processing for the dimensions and did not show signs of assigning higher weights to values.

Previous studies found that participants made decisions by separately comparing sums of money and delivery dates in the decision-making process of intertemporal choices (Arieli et al., 2011). In the information comparison process, the decision makers need to process information more within dimensions (rather than within options; Su et al., 2013; Zhou et al., 2019). This study shows when time and social distances are closer, alternative-based processing dominates. As the psychological distance increases, participants undertook more dimension-based processing. The psychological distance may affect the process of decision information search processing. This may imply that when the psychological distance is close, people have enough cognitive resources to evaluate the options. But when the psychological distance is too far, people will choose based on a single dimension in order to reduce the processing burden (Li et al., 2021).cc.

## 6 Limitations

The present study has the following limitations. Firstly, our study employed a money-based intertemporal choice paradigm, focusing primarily on the framework of profits. This approach might limit the understanding of intertemporal choices as it exclusively concentrates on financial gains (Zhao et al., 2015). Secondly, although similar discounting has been observed for real and hypothetical rewards (Johnson and Bickel, 2002), due to the “sign effect,” losses are discounted differently than gains, leading to varied decision-making processes in different life scenarios (Franco-Watkins et al., 2016; Ma et al., 2021). Further empirical data is needed to generalize our findings. Thirdly, our results indicate that an increase in probability distance does not significantly alter participants’ choice preferences. We suggest an interpretation rooted in the principles of uncertainty avoidance and attentional resources. It would be worthwhile to strictly investigate and establish the underlying mechanism of such effects. Future studies may further examine the interaction of psychological distance and task framework for different intertemporal choice outcome, the influence of time and amount parameters on decision-making, as suggested by previous studies (Sun and Li, 2010; Hardisty and Pfeffer, 2017).

## 7 Conclusion

Our research indicates distinct roles for various psychological distance dimensions in intertemporal decision-making. Greater temporal and social distances sway participants toward larger, delayed rewards, while increased probability distance does not significantly alter preferences. Additionally, as psychological distance expands, participants increasingly rely on a dimension-specific approach in their decision-making process.

## Data availability statement

The raw data supporting the conclusions of this article will be made available by the authors, without undue reservation.

## Ethics statement

The studies involving humans were approved by the Ethics Institutional Review Board of Shandong Drug and Food Vocational College. The studies were conducted in accordance with the local legislation and institutional requirements. The participants provided their written informed consent to participate in this study.

## Author contributions

YL: Conceptualization, Formal analysis, Writing – original draft. XC: Investigation, Methodology, Writing – review & editing.
